# Two Different Serum MiRNA Signatures Correlate with the Clinical Outcome and Histological Subtype in Pleural Malignant Mesothelioma Patients

**DOI:** 10.1371/journal.pone.0135331

**Published:** 2015-08-11

**Authors:** Monica Lamberti, Rosanna Capasso, Angela Lombardi, Marina Di Domenico, Alfonso Fiorelli, Antonia Feola, Alessandra F. Perna, Mario Santini, Michele Caraglia, Diego Ingrosso

**Affiliations:** 1 Department of Experimental Medicine, Section of Hygiene, Occupational Medicine and Forensic Medicine, School of Medicine, Second University of Naples, Naples, Italy; 2 Department of Biochemistry, Biophysics and General Pathology, School of Medicine, Second University of Naples, Naples, Italy; 3 Department of Thoracic Surgery, School of Medicine, Second University of Naples, Naples, Italy; 4 Department of Cardiothoracic & Respiratory Sciences, Second University of Naples, Naples, Italy; Istituto dei tumori Fondazione Pascale, ITALY

## Abstract

Pleural malignant mesothelioma (MPM) is a detrimental neoplasm affecting pleural sheets and determining a high rate of mortality. In this study, we have enrolled 14 consecutive patients (13 males and 1 female) with MPM (mean age: 70.3 ± 4.6 years). We have collected serum for the determination of a miRNA profiling using a low-density microarray real time PCR system in the serum of patients and comparing it with that one of 10 control counterparts affected by not-cancer-related pleural effusions. In the patients 5 miRNAs were up-regulated (miR101, miR25, miR26b, miR335 and miR433), 2 miRNA were downregulated (miR191, miR223) and two miRNAs were expressed exclusively in patients (miR29a and miR516). Based upon the changes in the expression of the above mentioned miRNAs we detected two distinctive miRNA signatures predicting histotype and survival in these patients: I) patients with more than 3/9 upregulated miRNAs or 3/9 upregulated miRNAs and miR516 not recordable or unchanged (signature A); II) patients with at least 3/9 downregulated or unchanged miRNAs and/or miR29a downregulated (signature B). Based upon these criteria, 5 patients were stratified in signature A and the remaining 9 in signature B. Patients with signature A had a significant shorter median survival than those with signature B (7 months vs. 17 months, 95% CI: 0.098–1.72, p = 0.0021), had a sarcomatoid or mixed histological MPM subtype and were diagnosed in stage II (3/5) and stage III (2/5). In conclusion, we suggest that miRNA signature A is predictive of sarcomatoid histotype and of worse prognosis in MPM.

## Introduction

MicroRNAs (miRNAs) are short non-coding RNAs, with gene regulatory functions at post-transcriptional level. Epigenetic mechanisms play a crucial role during development and differentiation; their deregulation have been also involved in aging and cancer-related processes [1,2]. The final amount of each microRNA can be regulated at the transcriptional or post-transcriptional level and it is well established that their expression depends on the activity of transcription factors or on epigenetic modifications, such as DNA methylation and histone modifications, that occur at their promoter sequences [3]. MiRNAs generally recognize, through base-pairwise complementary binding, specific seed sequences onto target mRNAs, thus triggering their degradation or by inhibiting their translation into proteins (the latter is the only mechanism in humans). Since miRNAs are involved in key functions, including differentiation and development, cell proliferation, stress response and apoptosis, alterations of miRNA expression have been early investigated and detected in various myeloproliferative and solid malignancies, thus immediately suggesting their involvement in disease onset and progression. One feature of various miRNA cluster is their involvement as oncogenes or tumor suppressor genes, as their aberrant expression has been reported to occur in colon, prostate, breast and lung cancers as demonstrated [4,5]. Identification and detection of differential miRNA arrays, between tumor and normal tissues, may provide early information on the neoplasm biological behaviour also leading to detect differentially expressed genes and pathways. In addition, characterization of miRNA signature within cancer tissues or biological fluids may represent biomarkers for early diagnosis and prognostic criteria, closely correlating with patient survival as well as serving as potential therapeutic targets [6]. Pleural malignant mesothelioma (MPM) is a highly malignant and resistant to therapy neoplasia, whose pathogenesis has been strictly related related to risk factors, particularly exposure to asbestos [7,8]. The latter is clearly related to triggering of inflammation mechanisms [9,10]. The three major MPM histopathologic subtypes (epithelioid, biphasic, and sarcomatoid) are clinically associated with different prognoses, although all characterized by poor survival, due to lack of both effective therapy and early prognostic biomarkers [11]. The association of definite miRNA expression patterns, endowed with prognostic meaning in MPM will be of great help to better manage therapeutic approaches of primary lesions and early detection of relapses. We present evidence of a differential signature of circulating miRNAs that is of potential prognostic meaning.

## Materials and Methods

### Patients

Fourteen consecutive patients (13 males and 1 female; mean age: 70.3 + 4.6 years) were included in the study at diagnosis and were subjected to videolaparoscopy and excisional biopsy of the pleura for histological examination. At the time of the enrollment, serum was collected and cryopreserved at -80°C. The patients were, thereafter, subjected to intervention or alternatively to chemotherapy. Other 10 patients (9 males and 1 female; mean age: 68.2 + 5 years) affected by not-cancer-related pleural effusions were enrolled and the sera were collected and used as normal controls. The study was approved by the Ethical Committee of the Azienda Universitaria Policlinico of the Second University of Naples (n. 103 on 12 January 2014) in compliance with the Helsinki Declaration. The informed consent for the participation to the study was approved and signed by the patients. A non MPM-serum pool was established as a reference control.

### miRNA Profiling in Serum Samples

Using TaqMan miRNA ABC Purification kit (Applied Biosystems), free circulating microRNAs were separated from 50μl of serum samples by magnetic beads, and miRNA expression was determined using the Megaplex Pool A Protocol and Megaplex preAmp protocol on microfluidic card type A (Applied Biosystems). Experiments were performed on Viia7 Thermalcycler (Applied Biosystems) and for each microfluidic card, the Ct of every miRNA was determined using Viia7 software (Applied Biosystems).

### Quantitative Real-Time PCR

Starting from 3μl of the serum free-circulating microRNA, selected miRNAs were evaluated with TaqMan MicroRNA Assays (Applied Biosystems) in individual patient serum samples. Relative quantification was performed using the ΔΔCt method using miR16 as housekeeping. Differential levels of each circulating miRNA were expressed, for each miRNA, as fold change level in each patient, with respect to the level of the same miRNA detected in the non-MPM control reference pool.

### Statistical evaluation of survival

Times to fatal outcome were the outcomes of interest. For patients who did not reach the end point, we censored time at the last follow-up visit. The Kaplan-Meier method was used to plot the probability of achieving the end point according to the two different miRNA expression signatures selected during the study. All time-to-event end points were analyzed using Cox proportional hazard regression models, and results were expressed as hazard ratio (HR) and 95% CI. Similarly, we also analyzed the same data with Gehan-Breslow-Wilcoxon test [12].

## Results

### Patient Characteristics

Fourteen consecutive MPM patients (13 males and 1 female; mean age: 70.3 + 4.6 years) were enrolled at diagnosis at the Division of Chest Surgery of the Second University of Naples. As control counterpart the serum of 10 patients (9 males and 1 female; mean age: 68.2 + 5 years) affected by non cancer-related pleural effusions was used. The confirmed histological subtypes were the following: 3 sarcomatoid, 7 epithelial and 4 mixed MPM. Five patients (all epithelial subtypes) were at stage I at the diagnosis and were all subjected to intervention. Three patients were at stage II and the remaining 6 at stage III, respectively. Eight patients were still alive at the moment of the analysis (Table 1).

**Table 1:** Patient Characteristics and Survival.

Pazient n°	Age	Sex	Hystology	Intervention	Stage	Survival (Months)
1	71	M	Mixed	N	II	7+
2	73	M	Sarcomatoid	N	III	6+
3	67	M	Epithelial	Y	I	10
4	72	M	Epithelial	N	III	6
5	65	M	Epithelial	Y	I	11
6	63	F	Epithelial	Y	I	17+
7	75	M	Sarcomatoid	N	III	6+
8	74	M	Mixed	N	III	5
9	70	M	Mixed	N	II	7+
10	71	M	Epithelial	Y	I	15
11	59	M	Epithelial	Y	I	14
12	74	M	Mixed	N	III	7+
13	75	M	Sarcomatoid	N	II	11+
14	67	M	Epithelial	N	III	13+

### Determination of serum miRNA expression

**Fig 1:**
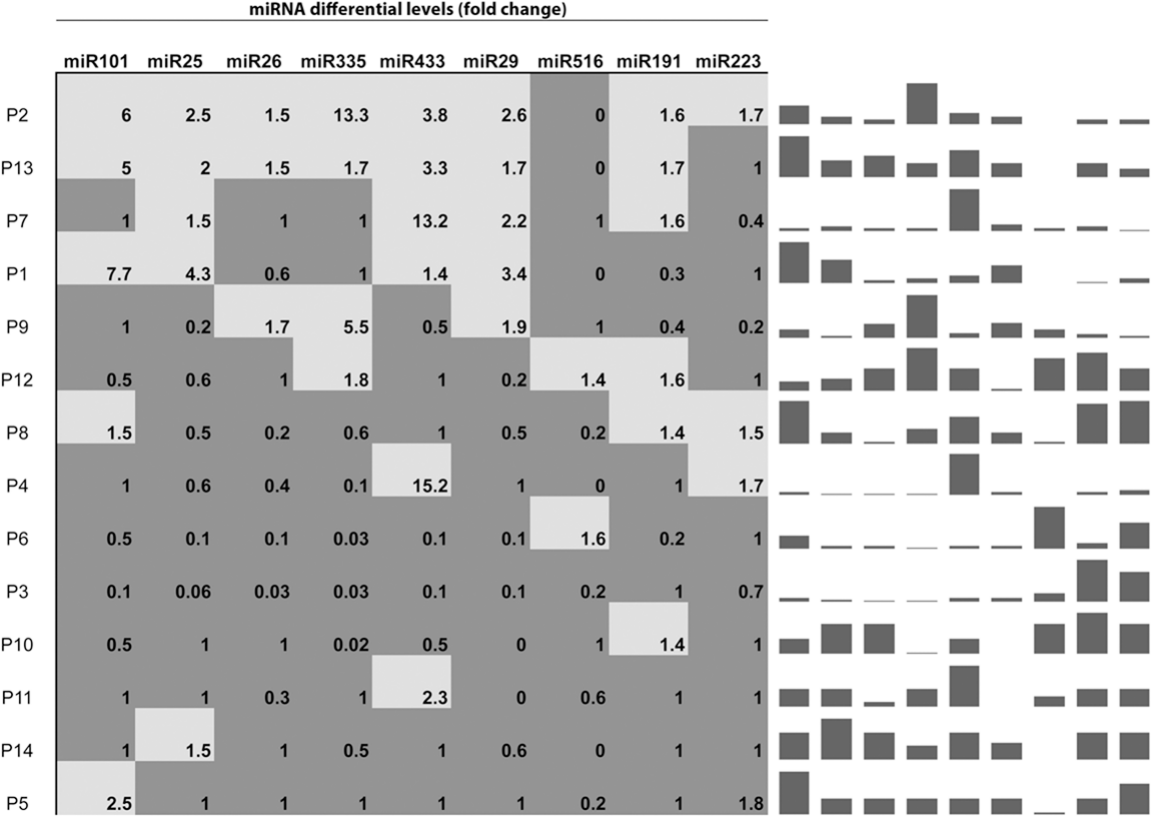
Serum miRNAs differentially expressed in the MPM patients, compared to non-MPM affected controls. Light gray boxes indicate upregulated miRNAs. Dark grey boxes indicate downregulated/no change/undetectable miRNAs. Spark-line graph refers to the corresponding fold-change differential levels for each miRNA and for each individual patient. Column height in each patient sample, in the spark-line graph, was internally normalized, to show relative expression of various miRNAs within the same signature.

**Fig 2:**
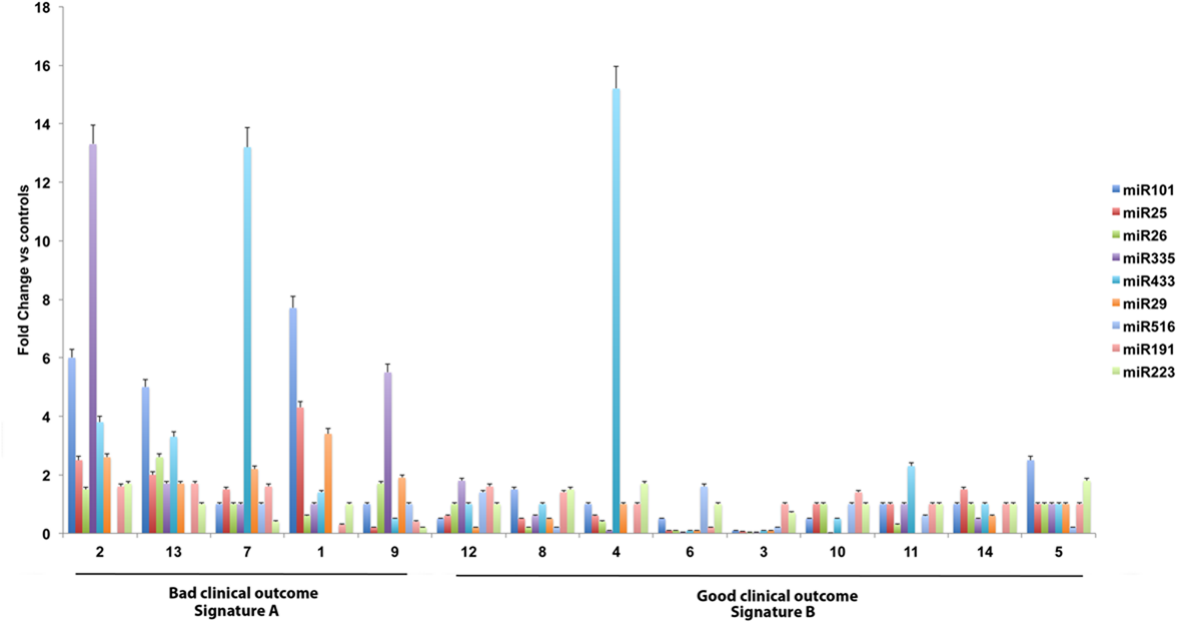
Differential miRNA levels in the two outcome-related MPM patient groups. Fold change level for each miRNA is represented. Color code to each miRNA has been assigned in order to uniquely identify each miRNA within individual serum samples. Analyses were performed and calculation accomplished as described under “Material and Methods”. Bars, standard deviations derived from at least three different calculations.

**Fig 3:**
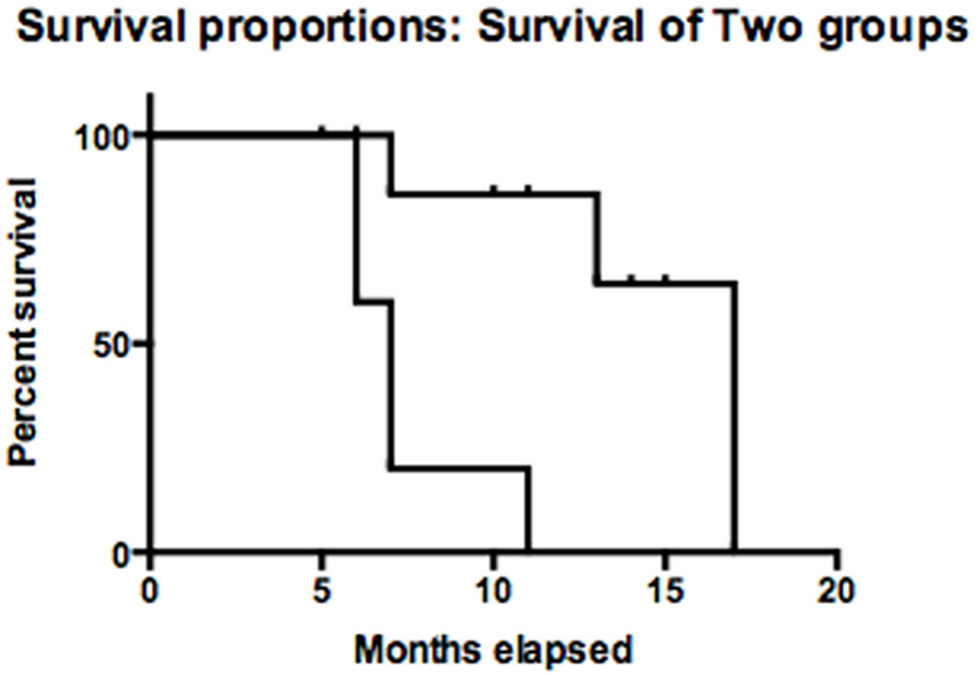
Kaplan-Meier estimate of the overall survival time of the patients stratified for miRNA signature A or miRNA signature B. P-values were derived from a log rank test (see “Materials and Methods”).

To determine specific miRNAs deregulated in serum samples of patients with MPM, we performed high-throughput miRNA expression profiles of serum samples using TaqMan microfluidic cards (Applied Biosystems). As a control, we assessed also serum samples collected from cancer-free subjects. Using microfluidic cards, 64 miRNAs were found expressed in control sera and 54 miRNAs were expressed in the samples from MPM patients. A subset of 30 miRNAs was found to be expressed in all samples. In this subset of miRNAs we found that in the patients 5 miRNAs were upregulated, miR101, miR25, miR26b, miR335 and miR433; 2 miRNA were downregulated, miR191, miR223 and two miRNAs were expressed exclusively in patients, miR29a and miR516. Quantitative real time PCR was performed to evaluate deregulated miRNAs identified in patients by miRNA expression profile in extended group of patients. Results are shown in (Fig 1) (Upregulated miRNAs are in light gray; Downregulated miRNAs are in dark gray or unchanged miRNAs or not detectable miRNAs). On the basis of this miRNA signature, we have subdivided the patients into two groups: I) patients with more than 3/9 upregulated miRNAs or 3/9 upregulated miRNAs and miR516 not recordable or unchanged (signature A); II) patients with at least 3/9 downregulated or unchanged miRNAs and/or miR29a downregulated (signature B) (Fig 2). Based upon these criteria, 5 patients (all males, median age: 73.0 years and mean age: 72.8 + 2.3 years) were stratified in signature A and the remaining 9 (8 males/1 female, median age: 67.0 years and mean age: 68.0 + 5.2 years) in signature B. Interestingly, the patients with signature A had a significant shorter median survival than those with signature B (7 months vs. 17 months, 95% CI: 0.098–1.72, p = 0.0021) (Fig 3). Moreover, all the patients with signature A were deceased at the moment of analysis and had a sarcomatoid (2/5 patients) or mixed (3/5 patients) histological subtype. On the other hand, for the signature B the most part of the patients (6/9 patients) were alive at the moment of diagnosis with epithelial (7/9 patients) or mixed (2/9 patients) histological subtype. In addition, 3/5 patients with signature A were diagnosed in stage II and the remaining 2/5 in stage III while 2/9 patients with signature B were diagnosed in stage III, 2/9 in stage II and 5/9 in stage I, respectively.

## Discussion

Aim of this research work was to detect peculiar circulating miRNA patterns, whose deregulation could be associated with more severe staging and, hence, predictive of, poor prognosis. We then analyzed circulating miRNA patterns in a group of MPM patients at various stages. We compared serum miRNA levels, between MPM patients and healthy controls and were able to identify different miRNA molecules consistently deregulated in these patients. Altered miRNA expression patterns were then validated by real time PCR. Therefore, we stratified the patients according to their histotypes and we recognized a miRNA signature, including three constantly overexpressed miRNAs, namely 25, 29 and 433, in the patient group showing poor prognosis, as well as other two miRNAs almost constantly overexpressed. Survival curves showed a clear association of overexpression of miRNAs 25, 29 and 433 with poor prognosis, compared with patients showing low circulating levels of the same miRNAs, thus allowing us to propose that 25, 29 and 433 may represent a circulating miRNA signature which is predictive of a poor clinical outcome. A study has recently demonstrated that miR25 is expressed in osteosarcoma cells, and it acts as oncogene by downregulating the cell cycle inhibitor p27, which is its direct target; tissue miR-25 expression was also associated with tumor progression and had prognostic implications in female lung adenocarcinoma patients and its upregulation is associated with poor prognosis in hepatocellular carcinoma patients [13,14]. MiR25 is overexpressed in ovarian cancer tissues by targeting LATS2 [15]. Moreover, *in vitro* miR25 overexpression promotes gastric cancer progression by downregulating transducer of ERBB2, 1 (TOB1) expression; consistently, patients with gastric cancer with high concentrations of miR25, in circulation, displayed poor survival [16,17]. On the other hand, the role of miR29b as a tumor suppressor or a tumor promoter, has been reported in several studies, depending on its targets in different tissues and cell types. For example, Zhang et al. [18] have demonstrated that miR-29 targets the TET genes, which is a key tumor suppressor frequently mutated in hematopoietic malignancies. Moreover, miR-29b, along with miR125b, miR29c, miR101, and miR7, is preferentially overexpressed in TET2-wild-type AML [19]. This is consistent with our results indicating the association of circulating miR101 upregulation with poor prognosis. On the other hand, miR29 can inhibit multiple myeloma cell proliferation via SP1 activation synergizing with bortezomib, causing a potent demethylating effect and inhibiting osteoclastosis and important pathogenetic effects in multiple myeloma patients [20,21,22]. An additional dysregulation spot concerned miR516, whose levels were undetectable or unchanged in the patient group with poor prognosis, if compared to healthy controls. MiR516a-3 was associated with higher aggressiveness of lymph node negative and estrogen receptor positive breast cancer and high metastatic potential to peritoneum in gastric cancer [23,24]. Other miRNAs were also downregulated in the group with poor prognosis, namely, miR101, miR26, miR433 and miR335. Our results suggest the conclusion that, as in other type of cancers, analysis of patterns of variations of circulating miRNA in MPM patients may be an effective way for identifying patient subsets with a very poor clinical outcome. The relevant downregulation signature pattern, reflecting corresponding dysregulated miRNA tissue expression, is particularly the one associated with low circulating levels of the subset including miRNA 25, 29 and 433. We also analyzed the pathways in which miRNA 25, 29 and 433 are involved, using the KEGG pathway map system (Table 2). Interestingly, we could identify a substantial number of pathways and relevant genes involved in many process regulating cancer pathophysiology, progression and resistance to therapy, such as: cell adhesion, oxidative metabolism, signal transduction, apoptosis. An interesting question regards the role of miRNA dysregulation as possible determinants of MPM aggressiveness and malignancy. In this respect, six microRNAs (miR21-5p, 23a-3p, 30e-5p, 221-3p, 222-3p, and 31-5p), when upregulated within tumor tissue, were significantly associated with a better survival curve [25]. In another study, miR31 upregulation in MPM tissue was associated with the worst outcome in sarcomatoid type MPM [26]. We found no correspondence of any of these tissue dysregulated miRNAs with the altered circulating patterns we detected. Häusler et al. assumed that cancer-induced miRNA profiles in cellular blood cells might already be detectable at early stages in the development of tumors, because it was shown that the formation of a pre-metastatic niche by hematopoietic cells is an early event of tumorigenesis and metastasis. Häusler et al. assumed that free circulating tumor-specific miRNAs in plasma or serum may be partly masked by high amounts of cellular miRNAs, but this loss of information is compensated by the information revealed from the cellular fraction. For ovarian cancer they believe, that stromal and myeloid progenitors or regulatory T cells, which are recruited to the tumor site, may significantly contribute to the miRNA profiles and the same might be true for MPM. Moreover, tumors can send immuno-suppressive and pro-angiogenic signals and induce the formation of pre-metastatic niches by hematopoietic cells that may shape miRNA profiles in blood cells. Based upon these indirect effects of tumors on immune and other circulating cells, the cellular fraction of human peripheral blood might be an appropriate source for biomarker discovery, even if miRNAs released from cancer cells become detectable in plasma or serum when a significant tumor mass has been accumulated [27]. However, at least at our knowledge, the present is the only study that has found a strong correlation between a serum miRNA signature and both histotype and prognosis of malignant pleural MPM patients. It is noteworthy that the non-invasive methodologies for the evaluation of molecular biomarkers in cancers are a pivotal issue in cancer research. Therefore, the identification of serum biomarkers detecting a subset of patients with poorer prognosis, could be paramount in the choice of the best treatment for MPM.

**Table 2:** KEGG pathways involving miRNA29, miRNA433 and miRNA25. Pathways involving gene targets for miRNA29, miRNA433 and miRNA25, as according to miRSystem software (ver. 20150312—mirsystem.cgm.ntu.edu.tw/). In order to draw inferences on potential functional interactions between miRNA and their gene targets, pathways identified are listed according to the KEGG (Kyoto Encyclopedia of Genes and Genomes) pathway map. Relevant nomenclature consists of a molecular network in terms of the KEGG Orthology (KO) groups. Genes are listed in each box according to their ability to serve as targets of each of the three miRNA considered in the upmost shaded headings. The miRNA targets involved in each specific pathway are reported according to a rank list where the first preferentially listed members, in each corresponding, box are common targets to more than one miRNAs.

Pathways	miR29	miR433	miR25
**FOCAL ADHESION**	CDC42—COL11A1—COL1A2—COL4A6—COL5A1—ITGA6—BIRC2—COL2A1—COL3A1—COL4A2—COL5A3—LAMA2 -	DOCK1—PAK4	CDC42—COL11A1—COL1A2—COL4A6—COL5A1—ITGA6 -
**AMOEBIASIS**	COL11A1—COL1A2—COL4A6—COL5A1—COL2A1—COL3A1—COL4A2—COL5A3—IFNG—LAMA2	RAB5C	COL11A1—COL1A2—COL4A6—COL5A1 -
**PARKINSON'S DISEASE**	ATP5G1—GPR37 -	COX6B1—COX8A	VDAC2
**UBIQUITIN MEDIATED PROTEOLYSIS**	FBXW7—BIRC2—BIRC6—PIAS4—SOCS1—UBA3	FBXW7—CDC27—TRIP12	FBXW7—CDC27—HERC2
**REGULATION OF ACTIN CYTOSKELETON**	CDC42—ITGA6—MYH9—PDGFC—WASF1	MYH9—DOCK1—FGF20—PAK4 -	CDC42—ITGA6 -CHRM5—IQGAP2—TMSB4Y -
**ANTIGEN PROCESSING AND PRESENTATION**	IFI30—IFNG	CREB1	CREB1
**CELL CYCLE**	STAG2—CDC7—YWHAE	CDC27—RAD21—STAG2	CDC27—RAD21—CDKN1C
**AXON GUIDANCE**	CDC42—EFNB3—ROBO1 EPHB3—RND1	PAK4	CDC42—EFNB3—ROBO1
**PATHWAYS IN CANCER**	CDC42—COL4A6—ITGA6—NCOA4—BIRC2—COL4A2—LAMA2—PIAS4	NCOA4—FGF20	CDC42—COL4A6—ITGA6—CEBPA—HHIP—TRAF3
**VASCULAR SMOOTH MUSCLE CONTRACTION**	MYL6	ADRA1A	ADCY3—ADM
**HUNTINGTON'S DISEASE**	ATP5G1 -	CREB1—COX6B1—COX8A	CREB1—VDAC2
**NEUROACTIVE LIGAND-RECEPTOR INTERACTION**	GRM4	ADRA1A	CHRM5—GRIA1
**SHIGELLOSIS**	CDC42—WASF1	DOCK1	CDC42
**VIRAL MYOCARDITIS**	LAMA2	MYH9	MYH9
	CDC42—WASF1	DOCK1	CDC42
**TIGHT JUNCTION**	CDC42—SPTAN1	MYH9	CDC42
**OOCYTE MEIOSIS**	YWHAE	CDC27	CDC27—ADCY3
**ENDOCYTOSIS**	CDC42—VPS25	CHMP5—RAB5C	CDC42—SMAD6
**T CELL RECEPTOR SIGNALING PATHWAY**	CDC42—IFNG	PAK4	CDC42
**MAPK SIGNALING PATHWAY**	CDC42—DUSP2—MAP2K6—TNFRSF1A	FGF20	CDC42—DUSP10—RPS6KA4
**RENAL CELL CARCINOMA**	CDC42	PAK4	CDC42
**PROSTATE CANCER**	PDGFC	CREB1	CREB1
**PROTEIN PROCESSING IN ENDOPLASMIC RETICULUM**	DNAJB11—SEC24D	DNAJB11—DNAJB12	DNAJB12—RRBP1
